# Improving knowledge of doctors and paramedics through effective bone procurement workshop: A cognitive approach

**DOI:** 10.1186/s12909-019-1685-9

**Published:** 2019-07-01

**Authors:** Suhaili Mohd, Norimah Yusof, Lee-Lee Lai, Md. Golam Hossain, Saravana Ramalingam, Suzina Sheikh AB Hamid, Wuey Min Ng, Azura Mansor

**Affiliations:** 1 Department of Orthopaedic Surgery (NOCERAL), Faculty of Medicine, University of Malaya, 50603 Kuala Lumpur, Malaysia; 2 Department of Statistic, University of Rajshahi, Rajshahi, 6205 Bangladesh; 3 Tissue Bank, School of Medical Science, Universiti Sains Malaysia, 16150 Kubang Kerian, Kelantan Malaysia; 4 Subang Jaya Medical Centre, 47500 Subang Jaya, Selangor Malaysia

**Keywords:** Bone allograft, Bone procurement, Transplantation, Tissue banking, Workshop training

## Abstract

**Background:**

Procurement of bone allograft must be performed by trained personnel. Improper handling and lack of knowledge during bone procurement will lead to contamination hence jeopardizing quality of the procured bones and expose bone recipients to risks of infection in post-operative phase. Bone procurement workshop is the fundamental training programme to enhance skill among personnel who has been or will be involved in bone procurement. This study evaluated the effectiveness of the workshop contents including teaching materials by assessing the knowledge on bone procurement among the participants before and after the workshop.

**Methods:**

Bone procurement workshop was held for 2 days for doctors and paramedics. The knowledge on bone procurement was evaluated in pre- and post-assessments by answering self administration questionnaire before and after the workshop, respectively.

**Results:**

A total of 50 participants comprised of doctors and paramedics attended the workshop however only 15 (55.6%) doctors and 12 (44.4%) paramedics completed the assessments. Overall, the mean total score for the post-assessment (61.4%) was significantly higher (*p* < 0.05) than that of the pre-assessment score (32.2%). The mean values of correct responses for the post-assessment was significantly higher (*p* < 0.05) than that of the pre-assessment in all five topics given during the workshop. The correct responses for the pre- and the post- assessments in the respective group of the doctors and paramedics were also statistically significant (*p* < 0.05). In the pre-assessment, the doctors had the highest score in Surgical Approach & Reconstruction (50%) while the paramedics had the highest score in Donor Screening & Selection Criteria (33.3%). In the post-assessment, the doctors had the highest score in Donor Screening & Selection Criteria (70%) while the paramedics in Packaging & Transportation (65.8%).

**Conclusions:**

The assessment managed to show that the workshop contents and teaching materials were effective in improving the cognitive knowledge of the personnel who would get involved in bone procurement under the National Donation Programme.

## Background

Bone grafting has been used in orthopaedic transplantations for more than hundred years [1]. Bone allograft acts as a substitute to bone autograft in orthopaedic reconstructive surgery mainly for providing mechanical support, repairing minor defects and replacing major bone loss in oncology cases [2–4].

Bone banking is still not popular among the orthopaedic surgeons despite the increasing usage of allografts in orthopaedic procedures lately [5]. Procedures of bone grafting involving bone procurement, graft preparation and delivery of bone allografts for transplantation is a complex and intricate process with varying practices among different bone banks [3]. Improper handling of procured bones might increase infection risks due to contamination [6].

In Malaysia, bone procurement must be carried out by surgeons namely orthopaedic surgeons under sterile condition. The procured bones are then properly packed by nurses or paramedics. Hence doctors and paramedics are the main players in procuring and storing bone allografts. Competency in procurement technique is vital in order to have high quality procured bone. Therefore they need to be given a special training to develop or improve skill in bone procurement before they are allowed to join bone procurement team in their respective hospitals or at national level. These well-trained personnel can also play an important role in the selection of suitable donors prior to bone procurement [7]. More donors will result in more bones to be procured thus improving bone stock to meet the increasing demand of bone allografts. Both orthopaedic surgeons and paramedics are therefore most eligible to assume the responsibilities of developing and managing a surgical bone bank [8].

Cadaveric bones must be procured in less than 12 h of time of death if the bodies are not refrigerated, and less than 24 h if refrigerated. Procurement is conducted either in operating theatre or mortuary. Preferably, procurement should be carried out immediately as colonization of bacteria will increase after 12 h [9].

Training on bone procurement must be able to provide the opportunity to develop surgical skills under a controlled environment and aseptic handling. By using cadavers, the hands-on session can simulate the actual bone procurement process, hence giving a real experience in handling human bodies. The training sessions would provide optimal opportunity for trainees to develop their competency in bone procurement procedures prior to the real practice and ability to reduce risks of bone contamination hence infection. Use of cadavers in surgical training has been practised for centuries and remains a highly regarded method of training due to the exposure to real anatomy and indeed anatomical variation [10]. Prior to hands-on practical session, a series of lectures were given to improve or enhance knowledge on bone banking, donor selection criteria, procurement technique, proper packaging and labelling.

Knowledge in tissue banking is still low in Malaysia. From our recent survey, only 12.5% of doctors and paramedic attended an orthopaedic conference were aware of the existence of local bone banks [11]. Malaysia has already conducted 2 bone procurement workshops namely in 2013 and 2015, in our effort to increase knowledge and develop more competent procurement team members. The contents of the workshops were further improved when the third workshop was conducted in 2017. Therefore the present study was aimed to assess the effectiveness of the bone procurement workshop, training programme and teaching materials, in improving knowledge for surgeons and paramedics.

## Methods

### Participants and workshop contents

Fifty participants comprising of 25 doctors and 25 paramedics were identified and selected by National Transplant Resource Centre (NTRC) and sponsored by the Ministry of Health Malaysia to participate in Bone Procurement Workshop 2017. The number of participants was limited by the working space and number of cadaverics available for the workshop. The participants came from various hospitals in Malaysia who have high potential to be involved in the bone procurement team of their respective hospitals. The selected participants already have basic knowledge in bone banking during their basic medical academic study. A number of them have been involved directly and indirectly in the National Donation Programme. The 2-day workshop comprised of seminar, forum and hands on session (Table 1). The teaching materials on bone procurement were designed in accordance to current guidelines on tissue banking by World Health Organization (WHO), Malaysian Tissue Act, Guidelines on Organ Tissue and Cell Transplantation Ministry of Health Malaysia, Standards of Asia Pacific Association of Surgical Tissue Banking (APASTB), Standards of American Association of Tissue Banks (AATB) and International Atomic Energy Agency (IAEA) Codes of Practice. The teaching modules were developed by the local experts in tissue banking and approved by Malaysian Association of Cell and Tissue Banking. Feedbacks to improve the contents were obtained from the academic staffs of Tissue Bank and Bone Bank in Malaysia, orthopedic surgeons, radiation experts and representatives from Ministry of Health. On Day 1, participants were given lectures related to Tissue Procurement Coordination Process (Lecture 1), Donor Screening & Selection Criteria (Lecture 2), Process Flow of Bone and Tissue Procurement (Lecture 3), Surgical Approach & Reconstruction (Lecture 4) and Packaging & Transportation (Lecture 5). Session for Day 1 ended with a forum on Medico-legal and Clinical Issues which was a compulsary for general knowledge. The issues included the current status of legal aspects and regulations on tissue banking. Hands-on practical class on Day 2 allowed the participants to conduct bone procurement, processing, packaging and labelling. Fresh frozen cadavers were retrieved from unclaimed bodies by the NTRC and they were kept at 4 °C prior to the workshop at Hospital Kuala Lumpur, Malaysia. Four cadavers were used where 6 doctors and 6 paramedics were stationed at each limb. After a demonstration by the main instructor, surgeons were guided to do bone procurement starting from upper to lower limbs. During the workshop, the paramedics were trained for packaging and labelling of the procured bones. Those activities were monitored by instructors who were orthopaedic surgeons and bone bankers. Discussion session on Day 2 covered all ascpects of bone procurement including bones procured from living donors.

**Table 1:** Format for Bone Procurement Workshop Sessions

Topic	Format	Duration (min)
Day 1		
Tissue Procurement Coordination Process	Didactic lecture	30
Donor Screening & Selection Criteria	Didactic lecture	20
Process Flow of Bone Procurement	Didactic lecture	30
Surgical Approach & Reconstruction	Didactic lecture	20
Packaging & Transportation	Didactic lecture/Demo	20
Medico-legal and Clinical Issues	Forum	60
Day 2		
Bone Procurement	Demonstration & video	90
Living Donor Programme	Discussion	
Bone Procurement, Processing & Packaging	Cadavers hands-on	180

### Pre and post workshop assessments

Workshop assessment comprised of three parts. Part 1 required participants to write their demographic information: name, gender; age, occupation, current working place and years in service. Part 2 contained questions on knowledge on tissue banking and tissue donation. Part 3 was assessment on bone procurement consisting of ten questions for each of five lecture topics which were presented during the workshop. The questions were multiple choice question where a “Yes”, “No” and “Not sure” were options for answer. This study was a pre-post quasi-experimental study to determine the difference score of two groups of participants i.e. doctors and paramedics, before and after attending the the workshop. The pre-assessment was conducted before the participants registered for the workshop. The post-assessment was conducted 2 weeks after the workshop allowing ample time for the participants to read lecture notes before answered the questions and to reduce recall bias among the participants. The post-assessment was administered online where participants were contacted via email and telephone. The assessment carried 10 marks for each question and percentage were calculated from the correct answers. A “No” and “Not sure” considered a single answer.

### Workshop evaluation

Workshop evaluation form was distributed at the end of the workshops to assess the impacts of the workshop, contents of the programme and lectures related to bone procurement. The participants were required to fill up an evaluation form and submitted immediately after the workshop ended. The workshop evaluation was based on Likert scale points from 1 to 5 with 1 = strongly disagree, 2 = disagree, 3 = neutral, 4 = agree, 5 = strongly agree. Free-form additional comment was also offered in the evaluation form.

### Ethical considerations

All the workshop participants were fully informed that the study was part of the bone procurement workshop. The purpose and the aims of the study were explained to them during registration at the registration counter. The participants were given consent form for declaration to give permission for their feedback and assessment scores to be used only in the study.

### Data analysis

All data were analyzed using SPSS software version 24.0 (IBM Corp. Armonk, New York). Demographic and knowledge of tissue banking were reported descriptively by frequency and percentage. Independent sample T-test was used to analyze the pre- and post-assessment scores by the participants within the group and between the two groups. Statistically significant threshold was set at *p* value ≤0.05.

## Results

### Socio-demographic characteristics

A total of 21 doctors and 19 paramedics responded to involve in the study which accounted as 84 and 76% of the total participants, respectively. Of these only 15 doctors (71.4%) and 12 paramedics (63.2%) completed the pre- and the post-assessment. Among the doctors, 9 were surgeons (33.3%) while others were medical officers (Table 2). In the paramedic group, 7 were medical assistants (25.9%) and the rest were nurses and medical laboratory technologists. Age range of the participants were 23 to 45 years old with male participants were dominant (70.4%). Most of the participants have been working more than 10 years in service (48.1%). The participants were mainly from government hospitals (85.2%).

**Table 2:** Socio-Demographic Characteristic of the Participants

Participants	n (%)
Doctor	15 (55.56)
Paramedic	12 (44.44)
Occupation	n (%)
Surgeon	9 (33.33)
Medical officer	6 (22.22)
Nurse	2 (7.41)
Medical Assistant	7 (25.93)
Medical Lab Technologist	3 (11.11)
Gender	n (%)
Male (%)	19 (70.37)
Female (%)	8 (29.63)
Mean/Median Age (range)	33.9/35 (23–45)
Years in service	n (%)
Less than 5 years	4 (14.82)
Between 6 to 10 years	10 (37.04)
More than 10 years	13 (48.14)
Current working place	n (%)
Government hospitals	23 (85.19)
Teaching hospitals	4 (14.81)

### Awareness in tissue banking

All participants as shown in Table 3 were aware about organ donation (100%). All doctors were aware about tissue donation compared to only 75% of the paramedic group. Again, all doctors knew about bone banking and only 58.3% of the paramedics knew about it. Majority of the doctors (86.7%) and half of the paramedics (58.3%) were aware about bone procurement. Majority of the doctors (66.7%) and the paramedics (75%) were never involved in bone procurement. Only 8 (29.6%) from the total participants have been involved in bone procurement for less than 5 times (Table 3).

**Table 3:** General Knowledge on Tissue Banking, n (%)

	Doctor	Paramedic		Doctor	Paramedic
Question 1. Do you know about organ donation?			Question 4. Do you know about bone procurement?		
Yes	15 (100)	12 (100)	Yes	13 (86.67)	7 (58.33)
No	0	0	No	2 (13.33)	2 (16.67)
Not sure	0	0	Not sure	0	3 (25.00
Question 2. Do you know about tissue donation?			Question 5. Have you ever involved in Bone procurement?		
Yes	15 (100)	9 (75.00)	Yes	5 (33.33)	3 (25.00)
No	0	2 (16.67)	No	10 (66.67)	9 (75.00)
Not sure	0	1 (8.33)	Not sure	0	0
Question 3. Do you know about bone banking?			Question 6. If yes, how many times?		
Yes	15 (100)	7 (58.34)	Less than 5×	5 (62.5)	3 (37.5)
No	0	1 (8.33)	More than 5×	0	0
Not sure	0	4 (33.33)			

### Bone procurement assessment

Based on correct responses, the participants in overall showed improvement in their scores, from only 32.2% in the pre-assessment to 61.4% in the post-assessment (Table 4). The improvement after the workshop in the total score as well as for all five topics of lectures were statistically significantly (*p* < 0.05). The doctors showed better performance than the paramedics in terms of the score from 40.1 to 64.8% while the paramedics’ score improved from 22.5 to 57.2% in the pre- and post-assessment respectively. The participants scored the highest correct responses (67.4%) after 2 lectures namely Donor Screening & Selection Criteria and Packaging & Transportation. The doctors scored the highest correct response in Donor Screening & Selection Criteria in the post-assessment (70%) compared to 43.3% in the pre-assessment; while the paramedics scored the highest correct response for Packaging & Transportation (65.8%) from only 22.5% in the pre-assessment. The lowest score in the pre-assessment recorded by the paramedics was in Surgical Approach & Reconstruction (12.5%) and significantly increased (*p* < 0.05) to 55% in the post-assessment. The doctors scored the lowest in Packaging & Transportation (32.6%) and significantly increased (*p* < 0.05) to 68.6% during the post-assessment. In the pre- assessment, the doctors had the highest score in Surgical Approach & Reconstruction (50%) while the paramedics in Donor Screening & Selection Criteria (33.3%). In the post-assessment, the doctors had the highest score in Donor Screening & Selection Criteria (70%) while the paramedics in Packaging & Transportation (65.8%). There were significant differences between the two groups (*p* < 0.05) either in the overall score or in each topic of lectures on bone procurement.

**Table 4:** Comparison of Correct Responses in Pre- and Post-assessments Scored by Doctors and Paramedics, Mean (%)

Topic	Overall			Doctor			Paramedic		
	Pre	Post	MD	Pre	Post	MD	Pre	Post	MD
Total score	16.15 (32.2)	30.70 (61.4)	14.55**	20.07 (40.1)	32.40 (64.8)	12.33**	11.25 (22.5)	28.58 (57.2)	17.33**
Tissue Procurement Coordination Process	2.85 (28.5)	5.26 (52.6)	2.41**	3.73 (37.3)	5.73 (57.3)	2.00**	1.75 (17.5)	4.67 (46.7)	2.92**
Donor Screening & Selection Criteria	3.89 (38.9)	6.74 (67.4)	2.89**	4.33 (43.3)	7.00 (70.0)	2.67**	3.33 (33.3)	6.42 (64.2)	3.09**
Process Flow of Bone and Tissue Procurement	3.19 (31.9)	6.00 (60.0)	2.81**	3.60 (36.0)	6.20 (62.0)	2.60**	2.67 (26.7)	5.75 (57.5)	3.08**
Surgical Approach & Reconstruction	3.33 (33.3)	6.11 (61.1)	2.78**	5.00/ (50.0)	6.60 (66.0)	1.60**	1.25 (12.5)	5.50 (55.0)	4.25**
Packaging & Transportation	2.81 (28.1)	6.74 (67.4)	3.93**	3.26 (32.6)	6.86 (68.6)	3.60**	2.25 (22.5)	6.58 (65.8)	4.33**

**: 5% level of significance (p < 0.05), MD: Mean difference

### Workshop evaluation

Evaluation on the workshop by the participants are summarized in Table 5. The participants mostly agreed with the listed impacts of the workshop when the means were greater than 4 for both the doctors and the paramedics. They also agreed that the workshop provided positive values to bone procurement. There were no statistically significant difference between the two groups in all aspects of the evaluation (*p* > 0.05). No additional comments were received from the participants.

**Table 5:** Evaluation of Bone Procurement Workshop using Likert scale points: 1 = Strongly Disagree, 2 = Disagree, 3 = Neutral, 4 = Agree, 5 = Strongly Agree

	Mean		*p* value
	Doctor	Para-medic	
Impact of the workshop			
Workshop objectives were clearly met	4.60	4.17	0.053
Enhanced my knowledge and skills	4.67	4.17	0.053
Procurement procedure was taught systematically	4.47	4.08	0.189
Information given is of practical value	4.40	4.00	0.170
Presentations (lectures) were useful	4.27	4.08	0.399
Questions were answered adequately during lectures and discussion	4.27	4.00	0.369
Questions related to Bone Procurement			
The workshop guided me through the experiential learning process	4.40	4.17	0.283
The workshop covered all the objectives of this session	4.27	4.08	0.465
The practical session of the workshop helped me to learn more effectively	4.27	4.00	0.321
The workshop has motivated and inspired me to actively be involved in bone procurement	4.13	4.25	0.661
The workshop has highlighted the important aspects of bone procurement	4.53	4.25	0.257
The workshop assessed my prior knowledge before attending the workshop	4.40	4.33	0.761
The workshop provided feedbacks about bone procurement which helped me to understand better	4.40	4.17	0.283
The workshop provided complete procedures for my future reference	4.00	4.25	0.369
The workshop extend my knowledge, understanding and skills in bone procurement procedures	4.47	4.33	0.500
The workshop helped me to learn in an organized, coherent and well-ordered manner	4.13	4.33	0.432

## Discussion

The study revealed that all of the participants were aware about organ donation but not all aware about tissue donation. The doctors were more aware about about bone banking and bone procurement than the paramedics. Future public awareness programme should emphasise on this supporting personnels.

In this study the scores for the correct response in the post-assessment were consistently higher than the pre-assessment indicating that the workshop managed to improve or enhance the knowledge of the participants. According to Beauchamps et al. (2016), the post-assessment rating would be significantly higher than the pre session rating when using integrated format modules including video, cadaveric training (hands on) and lectures [12]. Demographic factors in terms of gender, occupation, years of service and age of participants seemed not to influence the workshop assessments. Knowledge levels between the doctors and the paramedics were found to be inequal in this study. The doctors had higher scores in all topics of the pre-assessment with the highest score was for Surgical Approach & Reconstruction, which is a part of their nature of works. Surprisingly the highest score in the post-assessment for the doctors was in Donor Screening & Selection Criteria which indicated that the workshop managed to provide better understanding and made them more aware the importance of the inclusion and exclusion criteria for tissue donors in bone procurement. The workshop also improved their knowledge in bone packaging and transportation. The paramedics already had some knowledge in Donor Screening & Selection Criteria based on the score in the pre-assessment as they are the frontliner and directly deal with the donors. The highest score in post-assessment by the paramedics was in packaging and transportation indicating that the workshop managed to enhance their knowledge. The paramedics as the technical person are expected to continuously improve their know-how in proper handling of bones during the packaging and transportation to tissue bank.

The evaluation for the workshop in relation to the impacts of the workshop in enhancing the knowledge and skill in bone procurement by the doctors and the paramedics were similar. Both groups gave more than 4 indicating they agreed that the workshop contents were relevant and the programme was well organised to suit their expectations. The high rating given by the participants was so crucial to indicate that the workshop was succeded in improving their knowledge and assisting them to develop further their skills in bone procurement.

The assessments were able to evaluate the effectiveness of the bone procurement workshop. The training programme and teaching materials were relevant in improving knowledge and understanding in tissue banking while the hands-on practical class was essential in developing skill in bone procurement. In addition, the workshop provided a platform for the participants to discuss the current issues and to acquire new developments of tissue procurement in particular and tissue donation in general.

Cognitive knowledge on bone procurement was effectively assessed in the study and all aspects of bone procurement were mainly covered. In contrast, technical skill and surgical performance to perform bone procurement need to be assessed separately as more lengthy time and resources such as cadavers are required. Hence, this is the major drawback in the study where quality of the surgical outcome is not able to retrieve.

## Conclusions

The bone procurement workshop was effective in providing and improving knowledge of bone procurement among the medical personnels who have been and will be involved in the bone procurement. The assessment managed to quantify the improvement in selective subjects which are relevant to their respective tasks. Under the National Donation Programme, the workshop has been organised every 2 years to train the doctors and paramedics before they are appointed as the members of the national procurement team. We will continue to conduct the assessment on the workshop effectiveness and will conduct evaluation on the technical skill.

## Data Availability

Data and materials used in the study are available from corresponding author upon reasonable request.
